# Estimating the Dissolution of Anticancer Drugs in Supercritical Carbon Dioxide with a Stacked Machine Learning Model

**DOI:** 10.3390/pharmaceutics14081632

**Published:** 2022-08-05

**Authors:** Maryam Najmi, Mohamed Arselene Ayari, Hamidreza Sadeghsalehi, Behzad Vaferi, Amith Khandakar, Muhammad E. H. Chowdhury, Tawsifur Rahman, Zanko Hassan Jawhar

**Affiliations:** 1 Faculty of Industrial Engineering, South Tehran Branch, Islamic Azad University, Tehran 1584715414, Iran; 2 Department of Civil and Architectural Engineering, Qatar University, Doha 2713, Qatar; 3 Technology Innovation and Engineering Education Unit, Qatar University, Doha 2713, Qatar; 4 Department of Neuroscience, Faculty of Advanced Technologies in Medicine, Iran University of Medical Sciences, Tehran 1449614535, Iran; 5 Department of Chemical Engineering, Shiraz Branch, Islamic Azad University, Shiraz 7198774731, Iran; 6 Department of Electrical Engineering, Qatar University, Doha 2713, Qatar; 7 Department of Medical Laboratory Science, College of Health Science, Lebanese French University, Kurdistan Region 44001, Iraq

**Keywords:** artificial intelligence technique, ensemble model, anticancer solid drugs, solubility, supercritical CO_2_

## Abstract

Synthesizing micro-/nano-sized pharmaceutical compounds with an appropriate size distribution is a method often followed to enhance drug delivery and reduce side effects. Supercritical CO_2_ (carbon dioxide) is a well-known solvent utilized in the pharmaceutical synthesis process. Reliable knowledge of a drug’s solubility in supercritical CO_2_ is necessary for feasible study, modeling, design, optimization, and control of such a process. Therefore, the current study constructs a stacked/ensemble model by combining three up-to-date machine learning tools (i.e., extra tree, gradient boosting, and random forest) to predict the solubility of twelve anticancer drugs in supercritical CO_2_. An experimental databank comprising 311 phase equilibrium samples was gathered from the literature and applied to design the proposed stacked model. This model estimates the solubility of anticancer drugs in supercritical CO_2_ as a function of solute and solvent properties and operating conditions. Several statistical indices, including average absolute relative deviation (*AARD* = 8.62%), mean absolute error (*MAE* = 2.86 × 10^−6^), relative absolute error (*RAE* = 2.42%), mean squared error (*MSE* = 1.26 × 10^−10^), and regression coefficient (*R*^2^ = 0.99809) were used to validate the performance of the constructed model. The statistical, sensitivity, and trend analyses confirmed that the suggested stacked model demonstrates excellent performance for correlating and predicting the solubility of anticancer drugs in supercritical CO_2_.

## 1. Introduction

The low solubility of solid pharmaceutical substances in the aqueous-based media of the human body is often resolved by utilizing a higher dosage of drugs [1,2]. This increase in the dosage usually increases the cost of pharmacological treatment [3], decreases the drug’s therapeutic efficiency, and produces several side effects [2,4]. To overcome these critical limitations/drawbacks, synthesizing either micro- or nano-sized pharmaceutical substances with a uniform size distribution has been suggested by researchers [2,5]. Some researchers also used the ionically crosslinked complex [6] and self-indicating cellulose-based [7] gels to improver drug delivery. Therefore, a practical process must be established to synthesize pharmaceutical substances with these morphological characteristics.

Supercritical CO_2,_ which is a well-known solvent in the chemical [8], petroleum [9], polymer [10], energy [11], and food [12] industries, has also been successfully engaged in medical [13] and biomedical [14] engineering. The solubilities of stomach statin [15], malaria [16], Coronavirus [16], anti-inflammatory [17], antifungal [18], anti-hypertension [19], anticonvulsant [5,20], antibiotic [21], anti-prostatic tumor [5], antidiabetic [22], antiepileptic [23], and anti-cancer [24] drugs in supercritical CO_2_ have been experimentally measured/analyzed. These experimental investigations have often monitored the effect of temperature and pressure on the drug dissolution in supercritical CO_2_. Cancer is among the most deadly diseases known to humanity [25,26], but the solubility of anticancer drugs in supercritical CO_2_ is often low and ranges from 10^−7^ to 10^−3^ mole fraction. The literature has stated that measuring this low-scale property is expensive, time-consuming, and difficult [27].

Therefore, some researchers have utilized equations of state to simulate solid drug solubility in supercritical CO_2_ [28,29]. This technique relies on the solid drugs’ physio-chemical and critical properties to calculate their solubility in supercritical CO_2_ [2]. Unfortunately, equations of state not only require complex mathematical operations, but the required drug’s characteristics are also often unavailable [2].

Several empirical/semiempirical correlations have also been recommended to estimate the solubility of solid drugs in supercritical CO_2_ [30,31,32,33]. Although these correlations are easy to use and only require temperature, pressure, and solvent density to calculate the drug solubility value, they are often applicable for a specific system under predefined operating conditions [13]. Therefore, these methods cannot be applied to monitor the solubility of drugs in supercritical CO_2_ in a wide range of domains [13].

Recently, artificial intelligence models have been considered to estimate drug dissolution in supercritical CO_2_ as a function of operating conditions and solvent property [16,27,34,35]. The quantitative structure–property relationships [27], artificial neural networks [36,37], adaptive neuro-fuzzy inference systems [13,34], and support vector machines [37,38] have been applied to predict both drug and drug-like substances in supercritical CO_2_.

The stacked/ensemble models that are often constructed by systematically combining several previously designed machine learning (ML) models have found great popularity in different fields of science and technology [39,40,41]. This up-to-date modeling scenario has not previously been utilized to estimate anticancer drug solubility in supercritical CO_2_. Therefore, the current research combines the extra tree (ET), gradient boosting (GB), and random forest (RF) models to construct a reliable stacked model for estimating anticancer drug solubility in supercritical CO_2_. The suggested stacked approach can monitor the solubility of twelve anticancer drugs in supercritical carbon dioxide in a broad range of operating conditions. We can claim that the proposed model in this study is straightforward, easy to use, generalized, and has no applicable range limitation. Moreover, such an approach is essential for designing pharmaceutical processes using supercritical CO_2_.

## 2. Anticancer Drugs’ Solubility in Supercritical Carbon Dioxide

Laboratory investigations, empirical or semiempirical correlations, and machine learning models are often employed to measure or estimate the solubility of a specific solid drug in supercritical CO_2_ versus equilibrium pressure/temperature and solvent density. Since this study aims to design a single model for simultaneously estimating the solubility of twelve anticancer drugs in supercritical CO_2_, it is also necessary to include the solute property in the model development phase. Therefore, the machine learning models have been applied to extract the relationship defined by Equation (1).

(1) $$y_{drug}^{cal}\;=\;ML\;\left(M_{drug},\;T_{eq},\;P_{eq},\;\rho_{CO_{2}}\right)$$

It can be said that the machine learning methods are responsible for deducing the inherent relationship between the solubility ($y_{drug}^{cal}$) and drug molecular weight ($M_{drug}$), equilibrium temperature ($T_{eq}$) and pressure ($P_{eq}$), and solvent density ($\rho_{CO_{2}}$).

Table 1 introduces the experimental data gathered from the literature to develop the machine learning models. Moreover, Table 2 reports the molecular weight of the considered anticancer drugs and their chemical structure.

It should be mentioned that each row of Table 1 is actually a summary of multiple data instances, and that pressure, temperature, and CO_2_ density are input features. Moreover, an additional input feature is the molecular weight of drugs from Table 2, and the target feature to predict is anti-cancer drug solubility in supercritical CO_2_.

## 3. Methods

This study first develops three different ML regressors (i.e., extra tree, gradient boosting, and random forest) to monitor the equilibrium behavior of anticancer drug– supercritical CO_2_ systems. It then develops a stacked model using the three previously developed models as a base learner and linear regression as a meta learner.

### 3.1. Extra Tree

Geurts et al. [49] originally derived the extra tree regression (ETR) approach from the random forest (RF) algorithm [50]. According to the conventional top-down technique, the ETR develops a group of unpruned decisions (or regression trees) [49]. The ETR and RF models have two main differences. First, ETR utilizes whole cutting points and divides nodes by the random choice among these points. Second, it cultivates the trees utilizing the whole-learning samples to reduce bias as much as possible [49]. ETR controls the splitting process helping two parameters, namely, k and $n_{min}$. The former is the number of randomly chosen features in the node, and the latter represents the minimum sample size expected to separate nodes. In addition, k and $n_{min}$ determine the strength of both the selection of attributes and the average output noise, respectively. These parameters have a key role in improving the ETR accuracy and decreasing the possibility of overfitting [50]. 

### 3.2. Gradient Boosting

The gradient boost model (GB) is an ensemble regressor used to enhance accuracy of function approximation, according to the boosting process [51]. This scenario gradually reduces observed error by sequentially combining several weak learners. This study employs the decision tree as a weak learner. Although the performance of GB-based models depends on the loss function, the logarithm of loss function is often applied to handle regression problems. Furthermore, adaptive components and weak learners are the key parameters of GB-based models. If a gradient boosting model has 300 n estimators, it means that 300 decision trees (weak learners) have been coupled under the boosting process, and each tree is limited to 300 max depth. 

### 3.3. Random Forest

To perform a regression problem by the random forest (RF) method, the bootstrapping and bagging stages should be followed. The first stage generates a group of decision trees by the growth of each distinct tree that uses a random training dataset sample. The second stage breaks down the decision tree nodes after achieving the ensemble, where several random subdivisions of training samples are chosen during the initial bagging process. The decision-making is performed by choosing the best subdivision and its value [52]. In summary, the RF model can be viewed as a group of decision trees, in which $G\left(x,\theta_{r}\right)$ is the *G*th predicting tree and θ shows a uniform independent distribution vector assigned before the tree growth [53]. The Breiman equation (i.e., Equation (2)) is used to construct the forest (i.e., an ensemble of trees) by combining and averaging the whole trees [53].

(2) $$G\left(x,\theta_{1},\ldots \ldots \theta_{r}\right)=\frac{1}{R}\overset{R}{\underset{r=1}{\sum }}G\left(x,\theta_{r}\right)$$

### 3.4. Stacked Model

**Figure 1:**
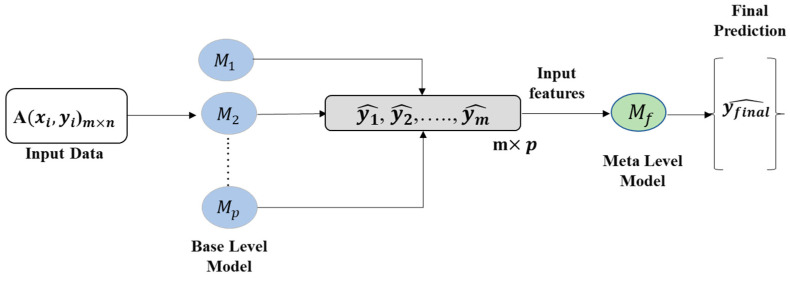
The general architecture of the stacked approach.

The study proposed a stacking-based approach and compared the performance with conventional ML regressors. This approach consists of a two-step learner such as base- learner and meta-learner. The three best-performing ML regression models were selected as base-learner models in the stacking model and linear regression was used as a meta- learner model ($M_{f})$ in the second phase of the stacking model and eventually produced the final prediction. Figure 1 shows the architecture of the proposed stacking model, which combined p numbers of best-performing regression models $M_{1},\ldots \ldots ,M_{p}$ using an input dataset A, with features ($x_{i}$) and corresponding label ($y_{i}$). In the first step, p numbers of base-level ML regression models produced the predictions $\hat{y_{1}},\ldots \ldots ,\hat{y_{m}}$. The predictions of the base learners were fed into the meta learner model ($M_{f}$) for the final prediction.

Algorithm 1 provides a step-by-step procedure to explain the construction of the stacked model.
**Algorithm 1.** The algorithm used for developing the stacked model
Input: training data
$A={\left\{x_{i},\;y_{i}\right\}}_{i=1}^{m}$
Output: a stacking regressor
$M_{f}$
1: Step 1: perform the training of the base-level regressors
2: for t = 1 to T do
3: Train
$h_{t}$
based on database of A
4: end for
5: Step 2: design new database of predictions
6: for i = 1 to m do
7:
$A_{h}=\left\{x_{i}{}^{\prime },\;y_{i}\right\}$, where
$x_{i}{}^{\prime }=\left\{h_{1}\left(x_{i}\right),\ldots \ldots ,h_{T}\left(x_{i}\right)\right\}$
8: end for
9: Step 3: perform the training of the meta-regressor
10: perform the training of
$M_{f}$
based on
$A_{h}$
11: return
$M_{f}$


It should be mentioned that the predictions of the level 0 model (base learner) are used as input to the level 1 model (meta learner). Moreover, the same set of training instances in the level 1 model (meta learner) and level 0 model are used (just with different features, obtained from base model predictions).

### 3.5. Performance Analysis

This study evaluates and compares the accuracy of the base and stacked machine learning scenarios using the *AARD*% (Equation (3)), *R*^2^ (Equation (4)), *MAE* (Equation (5)), *RAE*% (Equation (6)), and *MSE* (Equation (7)) indexes [54,55,56]. These indices quantify the deviation between the experimental solubility values ($y_{drug}^{\exp}$) and the calculated solubility data by the machine learning models ($y_{drug}^{cal}$).

(3) $$AARD\%=\;{\sum_{n=1}^{N}100\times \left(\left|y_{drug}^{\exp}-y_{drug}^{cal}\right|/y_{drug}^{\exp}\right)}_{n}/N$$

(4) $$R^{2}=\;1-\sum_{n=1}^{N}{\left(y_{drug}^{\exp}-y_{drug}^{cal}\right)}_{n}^{2}/\sum_{n=1}^{N}{\left(y_{{}_{drug}}^{\exp}-y_{{}_{drug}}^{ave}\right)}_{n}^{2}\;$$

(5) $$MAE=\;{\sum_{n=1}^{N}\left(\left|y_{drug}^{\exp}-y_{drug}^{cal}\right|\right)}_{n}/N$$

(6) $$RAE\%=100\;\times \;\;\sum_{n=1}^{N}{\left(\left|y_{drug}^{\exp}-y_{drug}^{cal}\right|\right)}_{n}/\sum_{n=1}^{N}{\left(\left|y_{drug}^{\exp}-y_{{}_{drug}}^{ave}\right|\right)}_{n}$$

(7) $$MSE=\sum_{n=1}^{N}{\left(y_{drug}^{\exp}-y_{drug}^{cal}\right)}_{n}^{2}/N$$

The above statistical criteria also require the average value of drug solubilities ($y_{drug}^{ave}$) and the number of data (*N*). Equation (8) defines the average value of drug solubilities in supercritical CO_2_.

(8) $$y_{{}_{drug}}^{ave}=\;{\sum_{n=1}^{N}\left(y_{drug}^{\exp}\right)}_{n}/N$$

Moreover, several graphical techniques (cross-plot, histogram, kernel density estimation, and Bland-Altman) and trend analyses have been applied to check the performance of the most accurate machine learning approach (i.e., the stacked model).

## 4. Results and Discussion

### 4.1. Developing Base Machine Models

The anticancer drug–supercritical CO_2_ phase equilibrium measurements (311 datasets) were randomly divided into internal and external groups (4:1 ratio). The five-fold cross-validation utilized the earlier group (i.e., 248 data samples) for the training and validation phases of the base learner machines. On the other hand, the remaining 63 data samples were engaged in the testing phase of the trained base-learner machines.

In this study, we leverage the hyperparameter optimization framework Optuna [57] as follows. First, the corresponding parameter spaces for the Scikit-Learn implementations of the RF, ETR, and GB models were identified. Then, the objective function (OF) was defined as the MSE. Lastly, a pipeline was applied to minimize the OF over a predefined maximum iteration on multiple cores (i.e., 300). Some of the RF and ETR hyperparameters, including the number of trees in the forest (i.e., number of estimators) were adjusted. We checked 80–150 trees in the forest and the best accuracy was obtained with 110 trees for the RF and 100 trees for ETR. Furthermore, upon checking different accuracy criteria (i.e., squared error, absolute error, and Poisson), squared error shows the best performance for both ETR and RF algorithms. In addition, the GB model [51] was tuned by the learning rate, ranging from 0.0 to 1 with a step size of 0.1. The results show that the learning rate = 0.2 produced the best performance. The model was also tuned with diverse loss functions (i.e., squared error, absolute error, Huber, and quantile). It was found that absolute error provided the model with the best performance. All reported results in this study were obtained by using the optimized models.

Table 3 introduces the uncertainty level observed in predictions of the base learner machines. The numerical values of five statistical indices are reported for the internal and external groups, as well as their combination. This table confirms that the deviation between the experimental solubility measurements and the associated predictions by the base-learner machines was relatively high. The extra tree model prediction accuracy was better than two other developed regression machines.

### 4.2. Designing the Stacked Model

As mentioned before, it is possible to build a stacked model by combining the previous three base learner machines utilizing the flowchart presented in Figure 1. Table 4 summarizes the prediction accuracy of the built stacked model in the cross-validation and testing phases and for all available datasets.

It can be concluded that the stacked model provides acceptable prediction accuracy for calculating the phase equilibrium behavior of the anticancer drug–supercritical CO_2_ binary system. The constructed stacked model estimated 311 data samples of anticancer drug solubilities in supercritical CO_2_ with excellent accuracy, i.e., *AARD* = 8.62%, *MAE* = 2.86 × 10^−6^, *RAE* = 2.42%, *MSE* = 1.26 × 10^−10^, and *R*^2^ = 0.99809. These values of the statistical indexes for predicting the ultra-low ranges of anticancer drug solubility in supercritical CO_2_ (10^−7^ to 10^−3^ mole fraction based on Table 1) are sufficient for designing pharmaceutical processes.

### 4.3. Comparison with the Other Modeling Scenarios

The literature has estimated solubilities of Decitabine [36] and Busulfan [38] in supercritical CO_2_ using adaptive neuro-fuzzy inference systems and support vector machines, respectively. These models have been developed to estimate the solubility of a single drug or two drugs in supercritical CO_2_, while our stacked model covers 12 different anti-cancer drugs. Table 5 shows that the accuracy of the stacked model is comparable or even better than the previously developed intelligent techniques.

This stage suggests a simple correlation based on the partial least-squares regression (PLS-R) to linearly relate the anticancer drug solubility in supercritical CO_2_ to the independent variables (Equation (9)).

(9) $$y_{drug}^{PLS-R}\;=\;\left(-1.66\;\times \;M_{drug}\;+\;2.62\;\times \rho_{CO_{2}}\;+\;21.16\times T_{eq}\;+\;9.62\times P_{eq}\;-\;9397\right)\;\times \;10^{-7}$$

The accuracy of the stacked model (*AARD* = 8.62%, *MSE* = 1.26 × 10^−10^ and *R*^2^ = 0.99809) is considerably better than the results obtained by the PLS-R (*AARD* >> 100%, *MSE* = 1.90 × 10^−8^ and *R*^2^ = 0.39307).

### 4.4. Evaluating the Performance of the Stacked Model Using Graphical Analyses

This section utilizes several graphical analyses to visually inspect the stacked model’s performance for predicting anticancer drug solubility in supercritical CO_2_.

**Figure 2:**
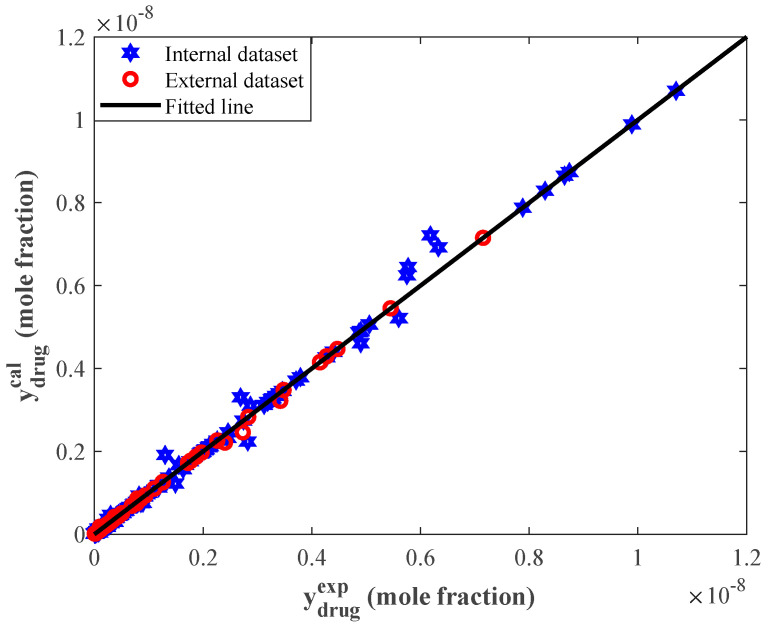
Correlation between experimental and calculated solubilities of the studied anticancer drugs.

Figure 2 depicts the calculated solubility values by the stacked model versus their corresponding experimentally measured values. The diagonal line shows those situations where predicted solubilities precisely coincided with their experimental counterparts (i.e., laboratory experiments equal prediction). The accumulation of both internal and external symbols in the vicinity of the diagonal line proved that the proposed stacked model successfully learned the equilibrium behavior of anticancer drug–supercritical CO_2_ systems.

Numerical values of the relative error (*RE*), average ($RE^{ave}$), and standard deviation (*SD*) have traditionally been used to evaluate the accuracy of a built model. Equations (10)–(12) present the mathematical expressions of the *RE*, $RE^{ave}$, and *SD*, respectively.

(10) $$RE\;=\;{\left(y_{drug}^{\exp}\;-\;y_{drug}^{cal}\right)}_{n}\;\;\;\;\;\;n=1,\;2,\;\ldots ,\;N$$

(11) $$RE^{ave}\;\;=\;\sum_{n=1}^{N}RE_{n}/N$$

(12) $$SD\;=\;{\left(\;\sum_{n=1}^{N}{\left(RE_{n}\;-\;RE^{ave}\right)}^{2}/N\right)}^{0.5}$$

**Figure 3:**
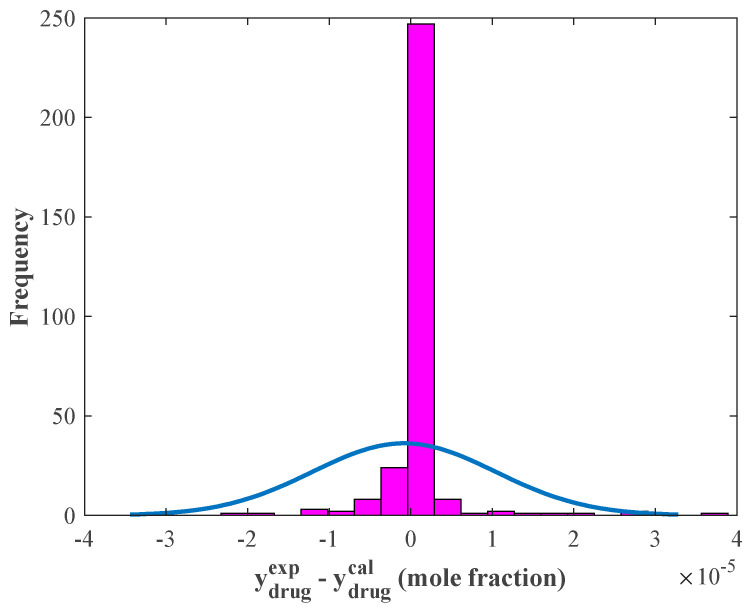
The histogram of residual errors provided by the stacked model (blue graph shows the normal distribution).

The histogram of the observed relative errors illustrated in Figure 3 justifies that the major part of the solubility data (248 samples) was estimated with a relative error equal to zero. Moreover, the relative errors’ average and standard deviation values were −8.2194 × 10^−7^ and 1.12 × 10^−5^ mole fractions, respectively.

**Figure 4:**
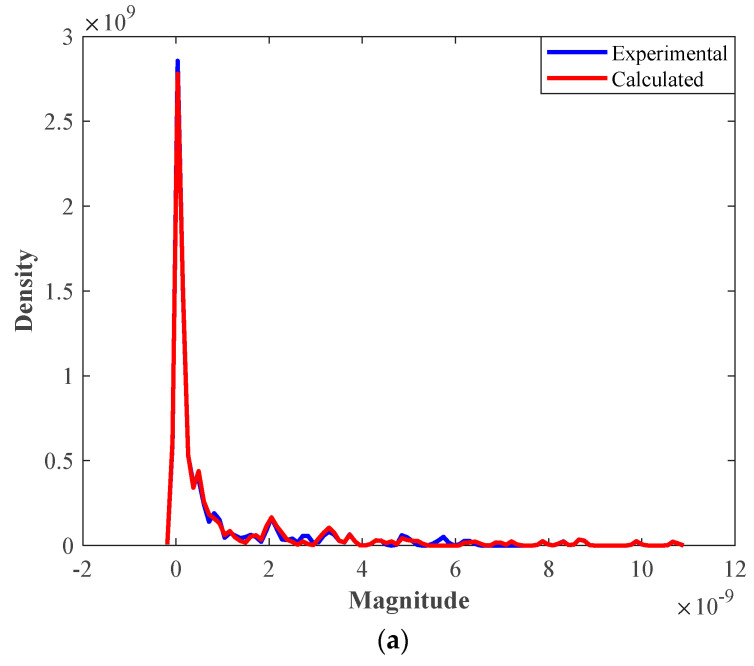

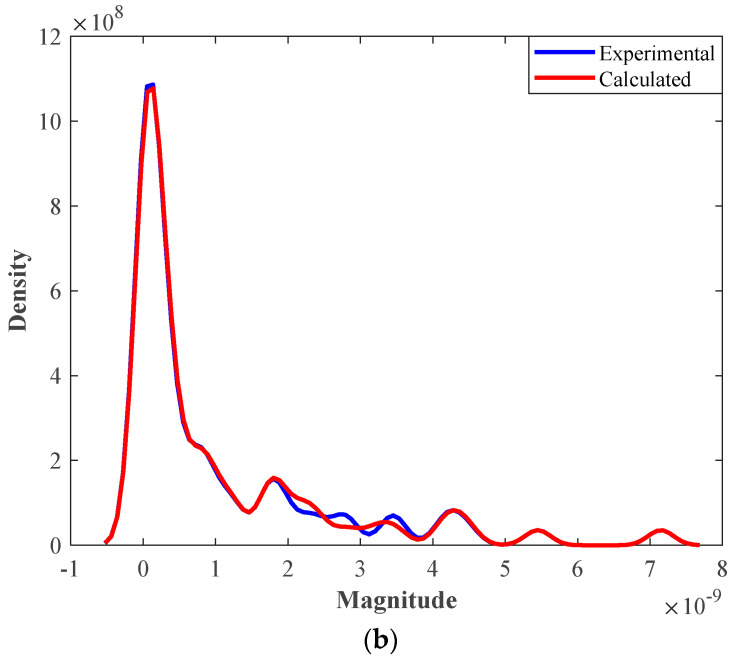
The kernel density estimation graphs for (**a**) internal and (**b**) external groups.

The kernel density estimation (KDE) graphs of the experimental and calculated solubility values for the internal and external groups are exhibited in Figure 4. The two graphs in the figure show that only a little deviation exists between the experimental and calculated KDEs of the external groups. This deviation is observable in the 2 × 10^−9^ < magnitude < 4 × 10^−9^ of Figure 4b.

**Figure 5:**
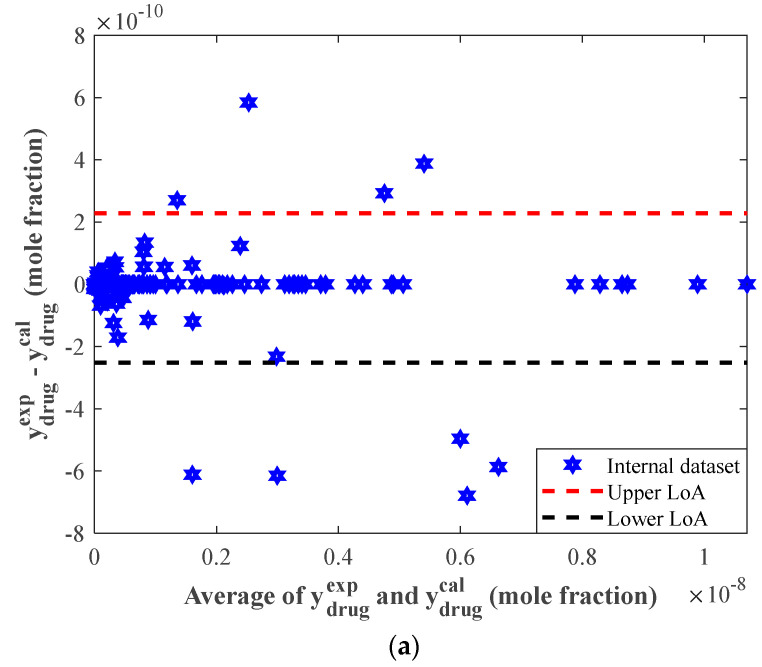

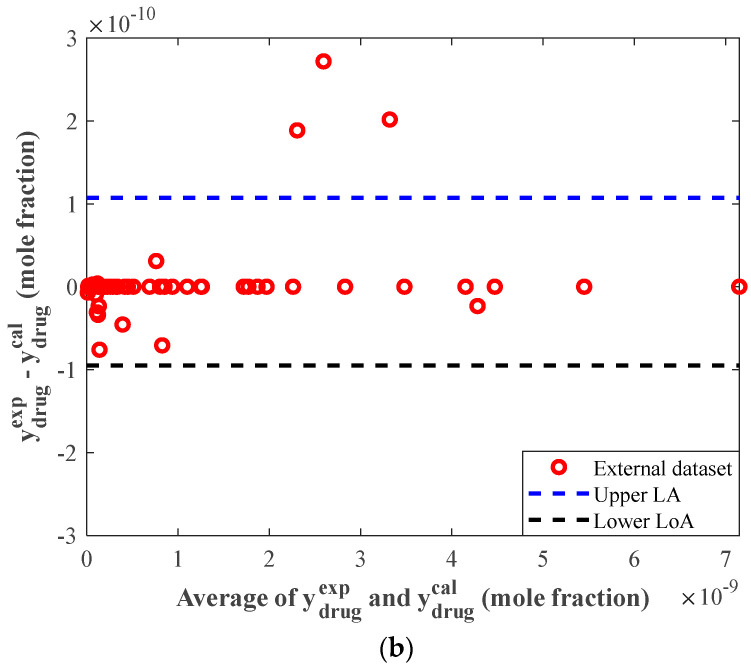
The Bland-Altman plots for (**a**) internal and (**b**) external groups.

Figure 5a,b depict the Bland-Altman plots for the internal and external anticancer drug solubility data. These figures have two horizontal dashed lines associated with the upper and lower *LoA* (95% limit of agreement). Equations (13) and (14) define the upper and lower *LoAs* indices, respectively [58].

(13) $$Upper\;LoA\;=\;+1.96\;\times \;SD\;+\;RE^{ave}$$

(14) $$Lower\;LoA\;=\;-1.96\;\times \;SD\;+\;RE^{ave}$$

The lower and upper *LoAs* in Figure 5a were −2.52 × 10^−10^ and 2.28 × 10^−10^, whereas Figure 5b had lower and upper *LoAs* of −9.51 × 10^−11^ and 1.07 × 10^−10^, respectively.

Figure 5a,b demonstrated that only 9 out of 248 internal data points (3.63%) and 3 out of 63 external data points (4.76%) were located outside the feasible domains.

### 4.5. Trend Analyses

This section investigates the effect of equilibrium temperature/pressure and anticancer drug type on the system behavior from experimental and modeling perspectives.

**Figure 6:**
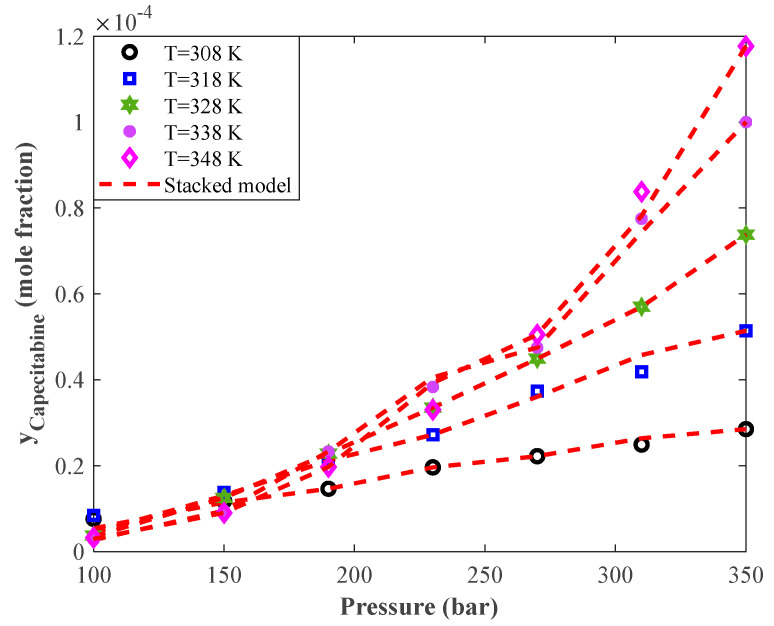
Monitoring the effect of pressure on the anticancer drug (Capecitabine) solubility in supercritical CO_2_ from the laboratory and modeling perspectives.

The profile of Capecitabine solubility in supercritical CO_2_ versus equilibrium pressure for five temperature levels was plotted in Figure 6. It can be seen that increasing the equilibrium pressure continuously improved the Capecitabine solubility in the applied supercritical solvent. It can also be concluded that the pressure effect on the solid drug solubility was linear at low temperature and became nonlinear at high temperature.

An excellent agreement between the laboratory-measured equilibrium data and the stacked model predictions is easily deduced from this figure. Indeed, the stacked model was trained so well that it precisely anticipated the effect of pressure/temperature change, linear/nonlinear behavior of the system, and all individual data points.

**Figure 7:**
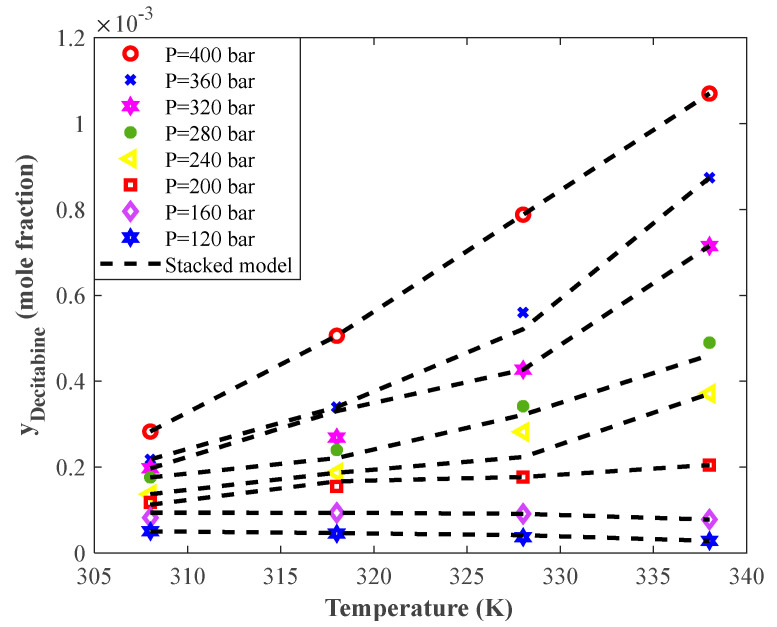
The experimental and modeling profiles of the effect of temperature on the solubility of the anticancer drug Decitabine in supercritical CO_2_.

Figure 7 exhibits the dependency of Decitabine solubility in supercritical CO_2_ on the temperature at eight pressure levels. This figure shows that the temperature had two different impacts on the solid drug solubility at low and high equilibrium pressures. Indeed, increasing the temperature at low pressures decreased Decitabine solubility in the supercritical CO_2_. On the other hand, increasing the temperature at high equilibrium pressures gradually intensified the Decitabine solubility in supercritical CO_2_.

The complete agreement between experimental solubility data and their associated stacked model predictions can also be justified in this figure. The stacked model correctly predicts the solubility–temperature profiles and accurately estimates all single data points.

**Figure 8:**
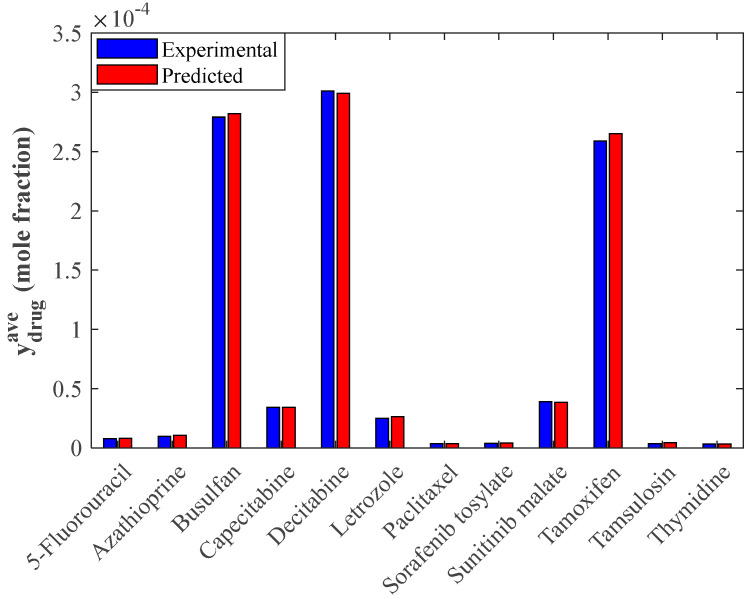
Average values of the solubility in supercritical CO_2_ of the studied anticancer drugs achieved from experimental data and modeling results.

Since the analyzed anticancer drugs have different compositions and chemical structures, their solubility is also influenced by drug type. The effect of anticancer type on the average solubility value is shown in Figure 8. As expected, the anticancer drugs show different dissolution tendencies in supercritical CO_2_. Decitabine, Busulfan, and Tamoxifen have the highest dissolution ability in the applied supercritical solvent. In contrast, Paclitaxel, Sorafenib tosylate, Thymidine, and Tamsulosin show the lowest tendency for dissolving in the considered solvent.

This figure also compares the average solubility values obtained by the experimental measurement and modeling analysis (i.e., stacked model). It is clear that the observed and calculated average solubility values are equal to up to four to five decimal places.

### 4.6. Importance of Independent Variables

As the last analysis, the Pearson technique [58] was applied to monitor the relative importance of each individual independent anticancer on the drug’s solubility in supercritical CO_2_. This technique presents a value ranging from −1 to +1 to clarify the direction and importance of the relationship between each pair of dependent and independent variables. Table 6 summarizes the results of this analysis.

## 5. Conclusions

The current research study applied the novel stacked model to precisely monitor phase equilibria of twelve anticancer drug–supercritical CO_2_ systems. The proposed stacked model was constructed by systematically combining extra tree, gradient boosting, and random forest machine learning models (known as base learners). Performance analyses of the based leaner models and the stacked model confirmed that the latter has the best accuracy in the cross-validation and testing phases. The designed stacked model showed excellent accuracy for predicting 311 experimentally-measured data samples (i.e., *AARD* = 8.62%, *MAE* = 2.86 × 10^−6^, *RAE* = 2.42%, *MSE* = 1.26 × 10^−10^, and *R*^2^ = 0.99809). This stacked model performance is far better than those results obtained by the base-learner model (i.e., extra tree, gradient boosting, random forest), machine learning approaches suggested in the literature (support-vector machines and Adaptive neuro-fuzzy inference systems), and partial least-squares regression (PLS-R). Moreover, the graphical accuracy monitoring techniques (cross-plot, histogram, kernel density estimation, and Bland-Altman) and trend inspections (solubility–pressure and solubility–temperature profiles) confirmed the reliability of the stacked model predictions. Finally, the experimental data and modeling results revealed that Decitabine and Thymidine have the highest and lowest tendency for dissolving in supercritical CO_2_, respectively.

## Figures and Tables

**Table 1:** Experimental data reported in the literature for the solubility of anticancer drugs in supercritical CO_2_.

Anticancer Drug	Pressure	Temperature	CO_2_ Density	Drug Solubility	No. of Data	Ref.
	bar	°C	kg/m^3^	Mole Fraction		
Sunitinib malate	120–270	35–65	388–914	5.00 × 10^−6^–8.56 × 10^−5^	24	[23]
Busulfan	120–400	35–65	383–971	3.27 × 10^−5^–8.65 × 10^−4^	32	[24]
Tamsulosin	120–270	35–65	384–914	1.80 × 10^−7^–1.01 × 10^−5^	24	[28]
Azathioprine	120–270	35–65	388–914	2.70 × 10^−6^–1.83 × 10^−5^	24	[42]
Paclitaxel	100–275	35–55	654–915	1.20 × 10^−6^–6.20 × 10^−6^	21	[43]
5-Fluorouracil	125–250	35–55	541–901	3.80 × 10^−6^–1.46 × 10^−5^	18	[43]
Thymidine	100–300	35–55	325–928	1.20 × 10^−6^–8.00 × 10^−6^	25	[43]
Capecitabine	152–354	35–75	477–955	2.70 × 10^−6^–1.59 × 10^−4^	35	[44]
Decitabine	120–400	35–65	383–971	2.84 × 10^−5^–1.07 × 10^−3^	32	[45]
Letrozole	120–360	45–75	319–922	1.60 × 10^−6^–8.51 × 10^−5^	20	[46]
Sorafenib tosylate	120–270	35–65	388–914	6.80 × 10^−7^–1.26 × 10^−5^	24	[47]
Tamoxifen	120–400	35–65	383–971	1.88 × 10^−5^–9.89 × 10^−4^	32	[48]

**Table 2:** Molecular weights and chemical structures of the investigated anticancer drugs.

Anticancer Drug	Molecular Weight	Molecular Structure
5-Fluorouracil	130	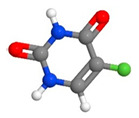
Azathioprine	277.26	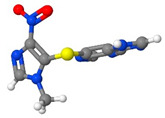
Busulfan	246.3	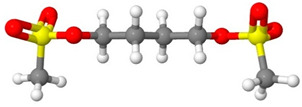
Capecitabine	359.35	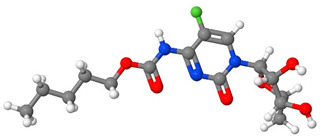
Decitabine	228.21	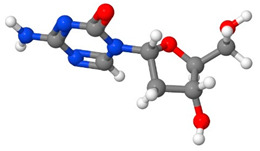
Letrozole	285.3	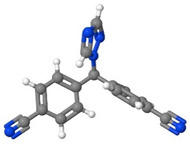
Paclitaxel	854	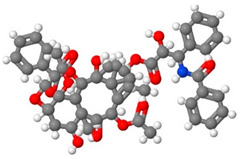
Sorafenib tosylate	637.03	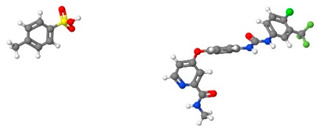
Sunitinib malate	532.56	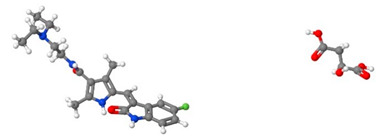
Tamoxifen	371.51	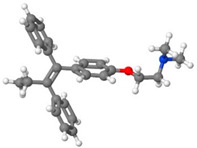
Tamsulosin	408.05	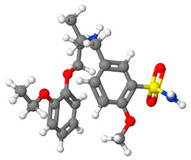
Thymidine	242	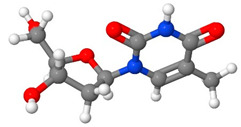

**Table 3:** Prediction accuracy of the base leaner machines.

Base Learner Model	Subgroup	*AARD*%	*MAE*	*RAE*%	*MSE*	*R* ^2^
Extra tree	Internal	11.52	9.21 × 10^−6^	7.71	1.19 × 10^−9^	0.98283
	External	37.44	2.58 × 10^−5^	22.85	2.36 × 10^−9^	0.95534
	All data	16.77	1.26 × 10^−5^	10.63	1.43 × 10^−9^	0.97838
Gradient boosting	Internal	21.04	1.57 × 10^−5^	13.13	1.40 × 10^−9^	0.97898
	External	43.07	2.48 × 10^−5^	21.99	1.97 × 10^−9^	0.95756
	All data	25.50	1.75 × 10^−5^	14.82	1.52 × 10^−9^	0.97560
Random forest	Internal	20.27	1.50 × 10^−5^	12.53	1.54 × 10^−9^	0.98354
	External	44.29	2.51 × 10^−5^	22.20	2.51 × 10^−9^	0.94926
	All data	25.14	1.70 × 10^−5^	14.38	1.73 × 10^−9^	0.97844

**Table 4:** Prediction accuracy of the stacked model.

AI Scenario	Subgroup	*AARD*%	*MAE*	*RAE*%	*MSE*	*R* ^2^
Stacked model	Internal	9.46	3.18 × 10^−11^	2.66	1.51 × 10^−20^	0.99791
	External	5.35	1.62 × 10^−11^	1.44	2.66 × 10^−21^	0.99946
	All data	8.62	2.86 × 10^−6^	2.42	1.26 × 10^−10^	0.99809

**Table 5:** Prediction accuracy of the stacked model.

Drug	Model	*R* ^2^	Reference
Decitabine	Adaptive neuro-fuzzy inference systems	0.99663	[36]
	Stacked model	0.99508	This work
Busulfan	Support vector machines	0.98327	[38]
	Stacked model	0.99054	This work

**Table 6:** Dependency of anticancer drug solubility in supercritical CO_2_ on the independent variables.

Information	Dependent–Independent Pairs			
	$y_{drug}\;-\;M_{drug}$	$y_{drug}\;-\;\rho_{CO_{2}}$	$y_{drug}\;-\;T_{eq}$	$y_{drug}\;-\;P_{eq}$
Pearson coefficient	−0.248	0.295	0.204	0.617
Direction of relationship	Indirect	Direct	Direct	Direct
Importance	Third	Second	Fourth	First

## Data Availability

The study data analyzed in this article can be obtained by request from the corresponding author.
